# Correction: Individual-based model highlights the importance of trade-offs for virus-host population dynamics and long-term co-existence

**DOI:** 10.1371/journal.pcbi.1011600

**Published:** 2023-10-27

**Authors:** Fateme Pourhasanzade, Swami Iyer, Jesslyn Tjendra, Lotta Landor, Selina Våge

The bacteriophage name B28 appears incorrectly throughout the article. The correct bacteriophage name is G28.
