# Supplementary material for: Individual-based model highlights the importance of trade-offs for virus-host population dynamics and long-term co-existence
Source: PLoS Comput Biol. 2022 Jun 8;18(6):e1010228. doi: 10.1371/journal.pcbi.1010228 (PMC9212155; doi:10.1371/journal.pcbi.1010228)
Supplement: S1 Pseudocode — The pseudocode describes details of initialization, interactions, budgets and evolutionary dynamics implemented in the model, step by step. (PDF) [file pcbi.1010228.s002.pdf]

Pseudocode for the IBM

// Initial host population
$\mathbf{H} \leftarrow \{h_1, h_2, \ldots, h_{N_h^0}\}$, where h_i has genotype $\{g_\alpha^0\}$ and mass $m_i \in [\frac{1}{2}, 1]$
// Initial virus population
$\mathbf{V} \leftarrow \{v_1, v_2, \ldots, v_{N_v^0}\}$, where v_i has genotype $\{g_\nu^0, g_\beta^0\}$
// Initial amount of dissolved phosphorus
$P_d^0 \leftarrow P - \Sigma_{i=1}^{N_h^0} m_i$
// Evolutionary dynamics of hosts/viruses
$P_d \leftarrow P_d^0$
for $t \in [1, T]$ **do**
  $P_d \leftarrow P_d + \omega(P_d^0 - P_d)$
  // Host dynamics
  for $h_i \in \mathbf{H}$ **do**
    // Loss due to washout
    if random() $< \omega$ **then**
      $\mathbf{H} \leftarrow \mathbf{H} \setminus \{h_i\}$
      continue
    end if
    // Loss due to mortality
    if random() $< \delta_h$ **then**
      $P_d \leftarrow P_d + m_i$
      $\mathbf{H} \leftarrow \mathbf{H} \setminus \{h_i\}$
      continue
    end if
    // Loss due to metabolism
    $\Delta m \leftarrow m_i \cdot \epsilon_h$
    $m_i \leftarrow m_i - \Delta m$
    $P_d \leftarrow P_d + \Delta m$
    // Growth
    $\Delta m \leftarrow \frac{\alpha g_\alpha P_d}{1 + \frac{\alpha g_\alpha P_d}{\mu_h}}$
    $m_i \leftarrow m_i + \Delta m$
    $P_d \leftarrow \max(0, P_d - \Delta m)$
    if $m_i > 1$ **then**
      // Host divides into two daughter cells, with possible mutation
      $g_\alpha \leftarrow g_\alpha$ gene of h_i
      if random() $< \pi_h$ **then**
        $g_\alpha \leftarrow$ Gaussian sample with $\mu = g_\alpha$ and $\sigma = \sigma_h$, truncated to [0, 1]
      end if
      $h_i^1, h_i^2 \leftarrow$ daughter cells of h_i, each with mass $\frac{m_i}{2}$ and genotype $\{g_\alpha\}$
      $\mathbf{H} \leftarrow \mathbf{H} \setminus \{h_i\} \cup \{h_i^1, h_i^2\}$
    end if
  end for

Pseudocode for the IBM (contd.)

```
    // Virus-host dynamics
    for $v_j \in \mathbf{V}$ do
        // Loss due to washout
        if random() $< \omega$ then
            $\mathbf{V} \leftarrow \mathbf{V} \setminus \{v_j\}$
            continue
        end if
        // Loss due to decay
        if random() $< \delta_v$ then
            $\mathbf{V} \leftarrow \mathbf{V} \setminus \{v_j\}$
            continue
        end if
        for $h_i \in \mathbf{H}$ do
            if random() $< \mathcal{C}(h_i, v_j)$ then
                // Virus compatible with host
                if random() $< \mathcal{V}(v_j)$ then
                    // Virus infects host and multiplies
                    $\mathbf{V} \leftarrow \mathbf{V} \setminus \{v_j\}$
                    $\mathbf{H} \leftarrow \mathbf{H} \setminus \{h_i\}$
                    $P_d \leftarrow P_d + m_i$
                    $g_\nu \leftarrow g_\nu$ gene of $v_j$
                    $g_\beta \leftarrow g_\beta$ gene of $v_j$
                    for $k \in [1, \kappa]$ do
                        if random() $< \pi_v$ then
                            $g_\nu \leftarrow$ Gaussian sample with $\mu = g_\nu$ and $\sigma = \sigma_v$, truncated to [0, 1]
                            $g_\beta \leftarrow$ Gaussian sample with $\mu = g_\beta$ and $\sigma = \sigma_v$, truncated to [0, 1]
                            $v_j^k \leftarrow k$th copy of $v_j$ with genotype $\{g_\nu, g_\beta\}$
                            $\mathbf{V} \leftarrow \mathbf{V} \cup \{v_j^k\}$
                        end if
                    end for
                end if
            end if
        end for
    end for
end for
```
