# Supplementary figures and images for: Individual-based model highlights the importance of trade-offs for virus-host population dynamics and long-term co-existence
Source: PLoS Comput Biol. 2022 Jun 8;18(6):e1010228. doi: 10.1371/journal.pcbi.1010228 (PMC9212155; doi:10.1371/journal.pcbi.1010228)

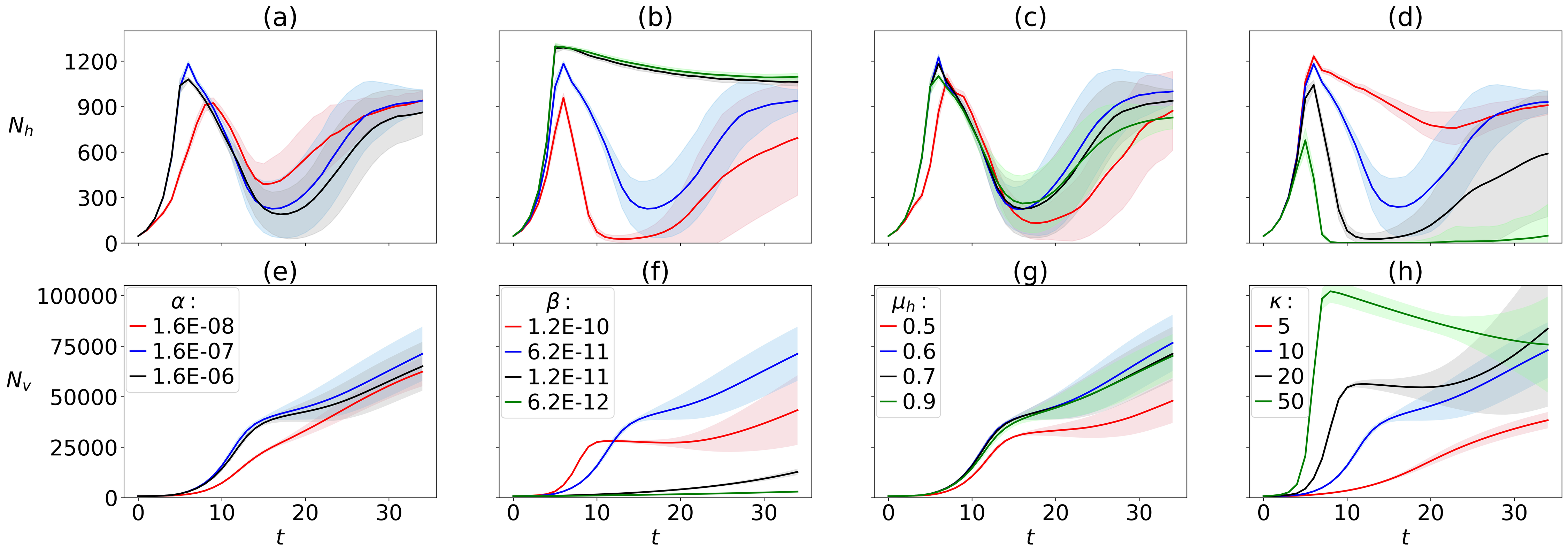

Supplement: S1 Fig — Host (a-h) and virus dynamics for various resource affinities for hosts (α), adsorption coefficients for viruses (β), maximum growth rates for hosts (μh) and burst size for viruses (κ). Plotted curves: ensemble averages from 100 runs for T = 36 h, with standard deviations shown as shading. (TIFF) [file pcbi.1010228.s003.tiff]

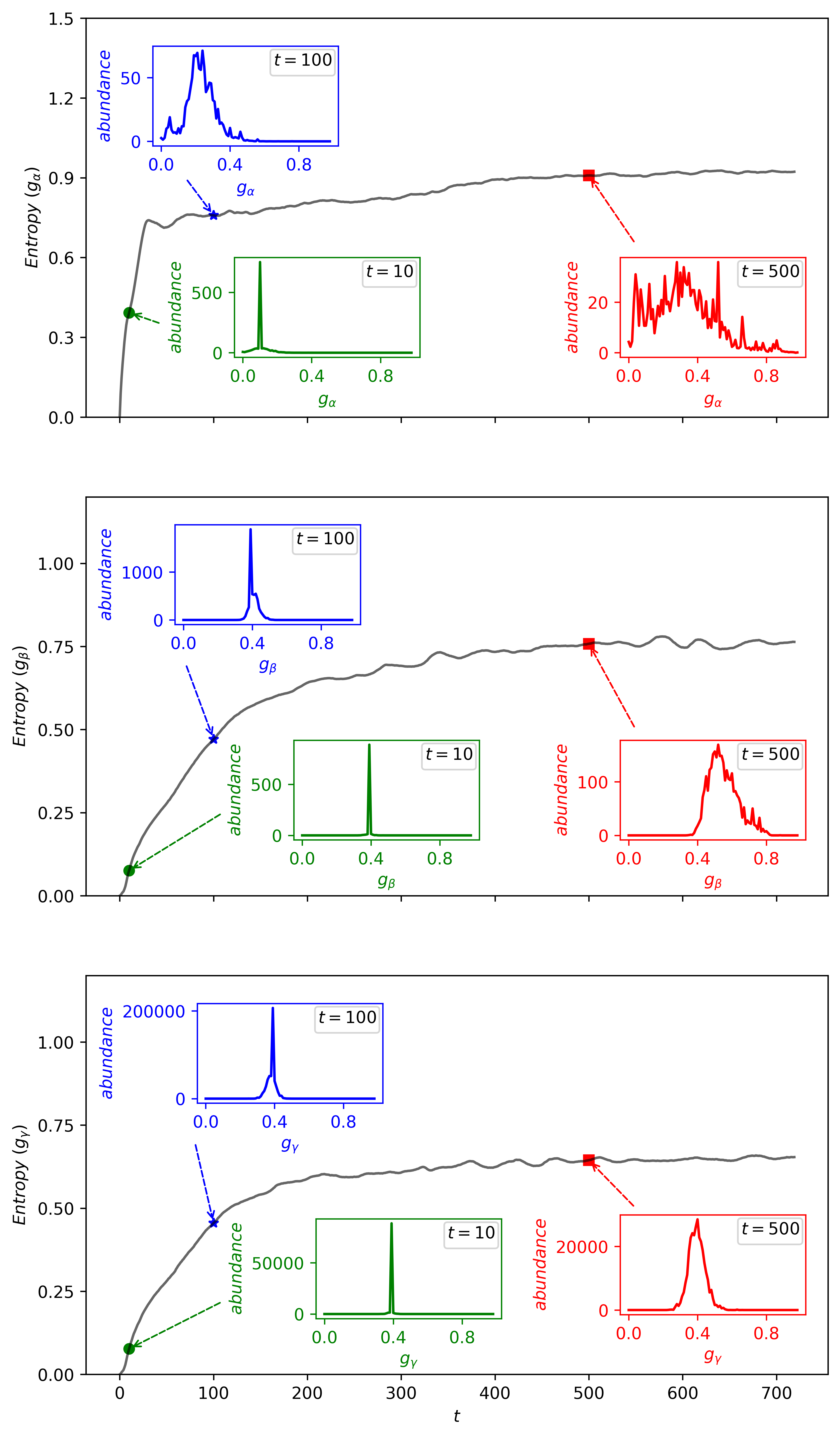

Supplement: S2 Fig — Diversity time-series based on Shannon entropy of ensemble averages from 100 runs in a trade-off based compatibiltiy and virulence scenario shown for a) nutrient affinity of host (gα), b) adsorption coefficient of virus (gβ), and c) memory gene of virus (gν). Small subpanels show abundance distribution for the genotypes at three distinct time points over the course of T = 720 h. (TIFF) [file pcbi.1010228.s004.tiff]
