# Supplementary material for: Individual-based model highlights the importance of trade-offs for virus-host population dynamics and long-term co-existence
Source: PLoS Comput Biol. 2022 Jun 8;18(6):e1010228. doi: 10.1371/journal.pcbi.1010228 (PMC9212155; doi:10.1371/journal.pcbi.1010228)
Supplement: Attachment — Submitted filename: PLOSCB.pdf [file pcbi.1010228.s005.pdf]

Impression

The paper presents an interesting idea and begins with a well-written, interesting abstract and introduction. It continues with interesting questions and experimentation and a nice coupling to motivating empirical data. Constraining the work to bacteria-phage interactions gives a nice chance for theoretical results to be tested more broadly across similar systems. The experiment of manipulating initial virus to host ratios is an interesting one. The paper sets up questions about tradeoffs shaping biodiversity, but most of the results shown are aggregated population dynamics (consistent with title), so there is a slight disconnect between the subtleties of the pitch and the results. By retooling (recalculating) a few key figures, it seems like new results are within reach.

Detailed comments

General

Based on the notation for **H**, I had a hard time imaging the model as individual-based. This may seem silly, but is it possible to give an explicit one sentence example of an **H** population vector (what are the contents - is it zeros and ones, is it the masses corresponding to those)? Ultimately **H** grows to be a vector of length 12-1600, correct? My initial impression (which I ultimately convinced myself was wrong) of, say h_2, was the biomass of individuals (plural) of 'genotype 2'. In the end I think I've pieced together that this is basically 'cell number 2', whether or not it remains part of the population at any time. I struggled a bit to not assume that the pairing h_2 with m_2, for example, was not the total biomass of *cells* of type number 2, but was the biomass of cell *number* 2. Again, I think a brief example might make this *more* clear.

Aside: Partly this is because the notation made me think each entry corresponded to a fundamentally different genotype. (This is probably all my fault as the reader, but since I spent a bit of time confused at first, I figured I better share.) Based on the fact that daughter cells genotypes could repeat, I assumed that entries in **H** corresponded to counts of each genotype (perhaps later weighted by their masses). I guess for a while I was missing something between the model notation and the implementation, perhaps that genotypes could be randomly assigned and unlikely to repeat exactly. Is the need to track individual cells (which makes this an IBM) preferable to aggregating cells by genome and considering this as a sort of stochastic metapopulation model (where the subpopulations are cells of a given genotype)?

Many of the parameters were drawn from [21] which seems to be a large ODE model. What I can't tell from the current model is if the time step is calculated as a result of the current state of the system (e.g., as in the Gillespie Algorithm) or if the time-step is fixed, and equal across the simulation. This raises some concern for the application of probabilities and rates to events happening in the interval. Without a more careful discussion of this ω (see below) can't be introduced as a rate, but used/described as a probability.

Model/Parameterization

The primary reference (source for parameters) is a differential equations model, so it seems that the parameters might not all transfer to the current work in a simple way. As an example ω is discussed in a few distinct ways (for reference, in [21] it is discussed as a dilution rate with unit 'per minute'): Table 1 - as a dilution rate, units 'per hour'; Line 71 - in an algebraic equation (without an obvious role as a rate); Line 75 - as a probability; and Line 89 - also as a probability.

Tying together the descriptions in a more careful manner will be necessary and will also help with interpretation of model mechanisms and its results.

Table 1 suggests that T is the total simulation duration, but throughout the paper (text, results, captions, figures) it seems like the authors discuss the length of simulations with respect to t. This is a bit tricky because the description of the model (Line 70) mentions "each time step $t \in [1, T]$". Does the model operate with a fixed time-step t (little-t), or is the the time-step variable? Is the implication that the model starts at $t = 0$, and then proceeds with a fixed, discrete time step of length one? Specific comments on that would help the reader better understand the implementation. Otherwise I would expect something like a continuous-time birth death process where the time step is large in small populations (less things can happen) and very small in large populations (because lots of things can happen, so they happen fast). The notation is complicated, but can this be summarized as

$$h_{i,n+1} = \ldots \text{something involving (death, division, growth) of } h_{i,n} \ldots.$$

Likewise, I feel like the evolution of P_d could really benefit from a concise presentation. It's tough to build it's dynamics from the separate chunks discussed across steps.

The mutation model suggests that novel genotypes are sampled from a normal distribution (itself valid on $(-\infty, \infty)$). Is there a truncation step applied to the new genotype value, or are genotypes rescaled to ensure they are in $[0, 1]$ (as Table 1 suggests)? Depending on the answer, would it not be reasonable to choose genotypes from a nicely parameterized distribution that is naturally defined on $[0, 1]$ (e..g, the Beta distribution is flexibly-shaped and defined on the interval).

Lots of changes to P_d are described, first the phosphorus update, then a few additions or subtractions depending on cell loss, growth, death. Is it possible to give a complete formula (see above, for $h_{i,n+1}$)? In terms of earlier comments about variable or fixed-length t, the mass gain described in Lines 79-80 suggests that the time step is fixed, otherwise I would expect mass gain (and other processes) to vary in magnitude or likelihood with t at each step. Can this be made more explicit? For example, if t does change, you might expect more growth over longer t and less growth over shorter t, so that the formula on Lines 79-80 would, again, depend on t. (Note: in the original ODEs this term wouldn't be expected to contain a t, but in the transition to a discrete-time framework it might (depending on details).

Line 108: If a host cell is infected, is it removed because it is assumed to die or because it can't be subsequently re-infected, and is no longer of interest dynamically? Are all bacteriophages lytic viruses (is that the implication)? Similarly, line 113 suggests that v_i represents the lone i^{th} virion, which is lost to infection, but by chance mutations at earlier stages, other identical viruses could exist in the population, right?

Clarifications

I assume upon extinction/mutations that the population vector $\mathbf{H}$ could shrink or grow in length? I'm interpreting N_h as a dynamic quantity that measures the size of the system (in terms of total number of individual cells)? Same for $\mathbf{V}$ which could grow in length to 160K?

Results

The results all show vertical axes of N_h and N_v which from Table 1 would be interpreted as total numbers of distinct cells. Is there any benefit to aggregating cells by genotype, or plotting measures of diversity?

Would it be helpful to quantify/present total diversity against time (if this isn't being what's shown) or biomass against time? Since this is discussed as a tool for understanding diversification and shaping diversity, the evolution of measures of diversity against time (even simple ones) might make for compelling vertical axes in graphs.

Presentation

Good attention is given to the presentation of the results in figures (in particular maintaining relatively consistent axes within and across figures, which facilitates comparison across results). I don't think the second panel of Fig 1, or either panel in Fig 2 are necessary. The formulas they present are relatively simple. Is there insight into whether the scale for the viral numbers is reasonable?

In my initial read, I missed the labels at the tops of columns in Figures 4-8, it would be nice if those could be moved into the figures, perhaps in legends. I think this would help consolidate information and make the reaction to the graphs more immediate.

It might be nice in the captions to include the baseline values of parameters not being varied (e.g., in Fig 4, what values of P and ω are used as VHR is being varied, and elsewhere, though I guess Line 162 addresses this). Also in Fig. 4, I'm not sure which panel(s) are being referred to with the phrase "Color codes for VHR match the codes in Fig. 3." since here there are more colors (and perhaps additional values) being plotted (the 3.30 line changes from orange to teal across figures, and a new 17.61 line appears).

Again note captions mention $t = 36$ but the Table suggests this should be $T = 36$. Figure 6 caption is missing formatting on y-axis (i.e., y-axis, see line 166).

Is it possible to create a flow/transition diagram for the model itself (terms are presented, but not pseudo-code/equations so some features are hard to 'add up'). For example, The P_d equation (Line 71) seems like it has a sort of logistic growth feel to it (the increment depends on ω and is proportional to the difference between initial and present values). How does that relate to the within step increment/decrement to P_d via host cell dynamics? Is there normalization to ensure at the end of each 'step' that the total amount of dissolved phosphorous isn't violated? Also, on Line 88 is virus washout accounting for free virus in the solution that is washed out, or viruses associated with (i.e., inside) host cells that are removed as *host cells* are washed out? The detailed accounting would help tell the complete story of the interaction(s) in a more convincing way.

Is the interesting result mentioned on line 200 a consequence of the formula on line 71 (large washout removes cells - phosphorus, but then P_d is small, so a large term proportional to $P_d^0 - P_d$ gets added back?).

Logistics and minor details

The Questionnaire suggests that code will be made available upon request. It would be a stronger commitment if it were posted to a public repository like GitHub or just directly as SI. Similarly no software language is referenced.

The sections **lines 115-126** and **Laboratory experiment** are pretty brief, though the lab section seems reasonable (to a reviewer that doesn't do experiments). I wonder if more quantitative description of the model investigation is warranted in the SI (this may already exist, but I didn't see SI with the submission). For example, is the sensitivity analysis done according to any particular procedure, or is it a comparison across conveniently selected parameter values (is sensitivity defined in any formal sense?)? In Line 118 'A range of ... probabilities', is there a justification or rationale

for the range tested, perhaps characterize the parameters of the experiments here explicitly rather than deferring to figure/captions for details.

One larger issue here is the normalization of parameters as experiments are conducted. it seems like the Table 1 value for VHR (17.61) is the max tested, but the value for P (71) is intermediate. Some sort of normaiization scheme would help with the interpretation of dynamics.

Line 101: consider a paragraph break at 'We also...' to enhance of the paragraph readability.

Line 113: change 'anymore' to 'any more'

Line 125: change 'parmeters' to 'parameters'

Line 147: change to 20% (no space)

Line 228: adsorption is misspelled

Line 284: typo, first words in line

Line 289: 'nutrient' not 'nutrientt' (two t's)

All figure captions: verify T or t for simulation durations (I think ti should be T).

Originality This seems like a fairly original attempt at implementing an existing approach to bacteria-phage dynamics, based on a published continuous time, ODE model. Specifically novel bacterial chemostat is presented in parallel with model results.

Innovation The paper addresses specific hypotheses about host-pathogen compatibility and virulence.

High importance to researchers in the field The model is quite parameter rich (parameters mostly drawn from earlier theoretical model). Without a bit more detail on interpretation and implementation of a few key steps, there is a bit of uncertainty that might limit use by others.

Significant biological and/or methodological insight Despite framing the paper around mechanisms of diversity, most of the results are summaries of dynamics. So some of the opportunity for insight is undercut by the choice of results. It seems possible to focus directly on the diversity/coexistence angle by aggregating hosts and phage across genotypes.

Rigorous methodology There are a couple inconsistencies on the use of parameters (rates vs probabilities) and a bit of missing detail on implementation (time-step). Addressing this would make the model more convincing. I didn't see reference to specific software used for implementation; this would facilitate reproducibility.

Substantial evidence for its conclusions Conditions under which coexistence is possible are illustrated. Answers to questions (diversity) alluded to in the framing of the paper seem within reach and could make a more robust story. It seems like this little bit of extra computation/analysis would add quite a bit to the current results.
