# Supplementary material for: Individual-based model highlights the importance of trade-offs for virus-host population dynamics and long-term co-existence
Source: PLoS Comput Biol. 2022 Jun 8;18(6):e1010228. doi: 10.1371/journal.pcbi.1010228 (PMC9212155; doi:10.1371/journal.pcbi.1010228)
Supplement: Attachment — Submitted filename: CoverLetter_ResponseToReviewers.pdf [file pcbi.1010228.s006.pdf]

Response to Reviewers

Dear editors of PLOS CB and reviewers.

We highly appreciate the three reviewers time and efforts to evaluate our work. The many detailed comments and suggestions are very useful, and we have addressed them all as best we could. We recognize that the model description needed more clarity and agree that including data on diversification strengthened our study. We also revised the figures according to the suggestions and provide full access to the model and data. Overall, our manuscript greatly improved with these changes, which we are grateful for. Our detailed responses to all the reviewers comments are given below.

Reviewer #1:

Pourhasanzade et al. present an interesting manuscript studying how bacteria-phage interactions mediate long-term coexistence of the bacteria and phage. The authors develop a discrete-time individual-based model that includes potential tradeoffs between bacterial growth rates and their ability to defend against infection by phage. They compare modeling results to those of simple infection experiments of E. Coli and a phage at different VHRs on plates.

The population dynamics of E Coli are shown in figure 3. For higher VHRs, the bacteria dynamics are interesting in that the bacteria initially increase then decrease (presumably due to the bacteria-phage interaction) before increasing yet again later on around hour 20. Under certain parameter regimes, the model successfully recapitulates the qualitative nature of these curves.

The individual-based simulations include bacterial growth, death, division, mutation, and virus-host interactions. Generally, I felt that the description of the model could be clarified; perhaps a simple schematic that illustrates the various steps of the model would help a reader understand each element of the model.

We thank the reviewer for the suggestion. We have included a pseudocode for the algorithm used in the IBM in the supporting information section, and we have added a reference to it in the text.

I was confused by a couple aspects of the model. I did not see the time step given (is it one hour?). A couple of issues: the parameter pi_h is the probability of mutation of host genotype—surely this must depend on the time step chosen. The parameters delta_h and delta_v are stated as mortality (or decay) rates with units per hour in table 1, but then in the text description (line 76 and line 89) these same parameters are treated as probabilities.

We thank the reviewer for pointing out this confusion. The issue arises from the fact that rates are used to express continuous processes, whereas IBMs express discrete dynamics. Translating rates to probabilities per time steps works when units of rates and units in time steps match. If time steps are of lengths of 1 hour and rates in units of h^{-1}, then statistically

speaking, the IBM will give correct results when interpreting rates as probabilities per time step. We added an explanation with this reasoning in the text and added a line in Table 1 to specify that time steps correspond to duration of 1 hour.

While the model is of course highly idealized, it is still quite complex and contains a large number of parameters (table 1). Many of these parameters are fixed at very specific values given by the study in reference 21. While some sensitivity analyses were performed for a few parameters, it is difficult to know whether model results are specific to this system, or whether results are more general. A broader exploration of parameter space may clarify this.

We thank the reviewer for pointing this out. We performed sensitivity analysis of most model's parameters but to keep focus, included only a selection in the text that shows influence of environmental (Fig. 4) and evolutionary (Fig. 5) mechanisms on population dynamics. Results showing sensitivity to host nutrient affinity, maximum growth rate, viral adsorption coefficient and burst size are now included in the supporting information section for the interested reader. With this we hope to keep the manuscript concise and accessible for general readers. Please note that the exact choice of parameter, over the test period of time, did not change the results in any marked way, suggesting that the results are qualitatively robust.

A major component of the model is the inclusion of bacterial evolution. When a cell divides, its genotype changes with some probability. These genotypes determine the compatibility of the virus and the bacteria through the function C_t. Yet it was difficult to see the connection to data, or to disentangle the effects of evolution from the innate structure of the model (the ecology) in determining population dynamics. Figure 3 shows E Coli population dynamics, but I did not see any sequencing or anything to show whether these dynamics are driven by evolution on the time scale of the experiment. It is difficult to know to what extent evolution is driving these dynamics in the experiments. Moreover, all results in figs 4-6 show population dynamics over time. It is difficult to know to what extent evolution is driving these dynamics in the model. Also, phage population dynamics were not measured, so these cannot be directly compared to the results of the model.

These are good points and we agree with the reviewer that it is difficult to say with certainty whether any evolutionary change tok place in the laboratory experiment without knowing genotypes in the beginning and end of the experiment. Based on a duration of only 1-2 days for the experiments, it is however likely that observed dynamics in the laboratory are purely "ecological", i.e., no evolutionary change underlies the observed dynamics. The intention to compare laboratory results with initial phase of our model simulations was indeed to check whether ecological population dynamics emerging from our simulated virus-host interactions are realistic. To show that evolution played a role in our model's longer-term simulations (Fig. 6), we now include results in the supporting information section showing how the diversity in genotypes for both host and virus traits increases over time, as measured by the Shannon entropy.

Data on viral abundance over the course of time in the laboratory experiment are unfortunately not available and are generally more tedious to measure than host

abundance. Still, with the virus numbers we get in our model being within a reasonable order of magnitude, we are confident that the qualitative dynamics captured in both the host and virus population are reasonable.

Minor issues:

I would say that Nh and Nv are state variables and not parameters as stated in Table 1.

Thank you, we have called these state variables instead of parameters.

Typo on line 289 "nutrientt" instead of "nutrient"

Thank you, we have fixed the typo.

I thought the figures could be improved by adding labels to the legends so that the reader would know what is being varied without having to read the caption. Many of the figures are means of many replicates. It might be nice to shade to show the amount of variation in replicates (standard deviation or similar).

We thank the reviewer for this good suggestion. We have updated the figures based on the suggestion, including labels in the legends and standard deviations as cloud error bars, reporting the average of 100 simulations for each plot. We hope that these changes improve the quality of the figures and make it easier for readers to grasp the full extent of our results.

Reviewer #2:

Impression

The paper presents an interesting idea and begins with a well-written, interesting abstract and introduction. It continues with interesting questions and experimentation and a nice coupling to motivating empirical data. Constraining the work to bacteria-phage interactions gives a nice chance for theoretical results to be tested more broadly across similar systems. The experiment of manipulating initial virus to host ratios is an interesting one. The paper sets up questions about tradeoffs shaping biodiversity, but most of the results shown are aggregated population dynamics (consistent with title), so there is a slight disconnect between the subtleties of the pitch and the results. By retooling (recalculating) a few key figures, it seems like new results are within reach.

We thank the reviewer for sharing this impression. We address all comments below. Agreeing in particular on the potential of discussing diversity, we include results in the supporting information section showing a synthesis of evolutionary diversification in host and virus genotypes over time, using the Shannon entropy index. However, to focus the scope of this manuscript, we hope to prepare a more detailed analyses of effects on diversity in a separate study.

Detailed comments General

Based on the notation for H, I had a hard time imaging the model as individual-based. This may seem silly, but is it possible to give an explicit one sentence example of an H population vector (what are the contents - is it zeros and ones, is it the masses corresponding to those)? Ultimately H grows to be a vector of length 12-1600, correct? My initial impression (which I ultimately convinced myself was wrong) of, say h2, was the biomass of individuals (plural) of 'genotype 2'. In the end I think I've pieced together that this is basically 'cell number 2', whether or not it remains part of the population at any time. I struggled a bit to not assume that the pairing h2 with m2, for example, was not the total biomass of cells of type number 2, but was the biomass of cell number 2. Again, I think a brief example might make this *more* clear.

We thank the reviewer for bringing this up. **H** is a set representing the host population at time t. Each element h_i in **H** denotes a particular host having certain traits, such as, its genome and mass. The virus population is similarly represented by the set **V**. Yes, the sets **H** and **V** grow (and shrink) as the dynamics unfold. We have added a clarification on this in the model description to be more clear.

Aside: Partly this is because the notation made me think each entry corresponded to a fundamentally different genotype. (This is probably all my fault as the reader, but since I spent a bit of time confused at first, I figured I better share.) Based on the fact that daughter cells genotypes could repeat, I assumed that entries in H corresponded to counts of each genotype (perhaps later weighted by their masses). I guess for a while I was missing something between the model notation and the implementation, perhaps that genotypes could be randomly assigned and unlikely to repeat exactly. Is the need to track

individual cells (which makes this an IBM) preferable to aggregating cells by genome and considering this as a sort of stochastic metapopulation model (where the subpopulations are cells of a given genotype)?

We thank the reviewer for the question. We decided to track individual cells instead of aggregating them by genome because as a future line of research, we want to study the virus-host interaction pattern and the diversification of their genome in considerable detail. By being more explicit in the text about the nature of **H** and **V** being sets that grow and shrink as dynamics unfold, we hope to avoid utter confusion.

Many of the parameters were drawn from [21] which seems to be a large ODE model. What I can't tell from the current model is if the time step is calculated as a result of the current state of the system (e.g., as in the Gillespie Algorithm) or if the time-step is fixed, and equal across the simulation. This raises some concern for the application of probabilities and rates to events happening in the interval. Without a more careful discussion of this ω (see below) can't be introduced as a rate, but used/described as a probability.

We thank the reviewer for raising this concern. The time step in our model, expressed in hours (h), is fixed across the simulation. The value is set to 1 h in our simulations. Since the time steps are of unit length and rates are in units of h^{-1}, we are justified in interpreting rates as probabilities; our discrete-time model, in a statistical sense, will produce the same results as a continuous-time model involving rates.

We added an explanation with this reasoning in the text and added a line in Table 1 stating that time steps correspond to 1 hour.

Model/Parameterization

The primary reference (source for parameters) is a differential equations model, so it seems that the parameters might not all transfer to the current work in a simple way. As an example ω is discussed in a few distinct ways (for reference, in [21] it is discussed as a dilution rate with unit 'per minute'): Table 1 - as a dilution rate, units 'per hour'; Line 71 - in an algebraic equation (without an obvious role as a rate); Line 75 - as a probability; and Line 89 - also as a probability. 1 Tying together the descriptions in a more careful manner will be necessary and will also help with interpretation of model mechanisms and its results.

Please see comment above and explanation now given in the text (since our time steps have duration of "one hour", and unit of the rate is "per hour", rates in individual-based model equations can be treated as "probabilities per time step").

Table 1 suggests that T is the total simulation duration, but throughout the paper (text, results, captions, figures) it seems like the authors discuss the length of simulations with respect to t. This is a bit tricky because the description of the model (Line 70) mentions "each time step t ∈ [1, T]". Does the model operate with a fixed time-step t (little-t), or is the the time-step variable? Is the implication that the model starts at t = 0, and then

proceeds with a fixed, discrete time step of length one? Specific comments on that would help the reader better understand the implementation. Otherwise I would expect something like a continuous-time birth death process where the time step is large in small populations (less things can happen) and very small in large populations (because lots of things can happen, so they happen fast). The notation is complicated, but can this be summarized as hi,n+1 = . . .something involving (death, division, growth) of hi,n . . . . Likewise, I feel like the evolution of Pd could really benefit from a concise presentation. It's tough to build it's dynamics from the separate chunks discussed across steps.

We thank the reviewer to point out this confusion. The time-step is fixed to one hour, which we now state explicitly in the text, and to be clear we also included t in the table, specifying it as a symbol for time step duration with given value and unit.

The mutation model suggests that novel genotypes are sampled from a normal distribution (itself valid on (−∞, ∞)). Is there a truncation step applied to the new genotype value, or are genotypes rescaled to ensure they are in [0, 1] (as Table 1 suggests)? Depending on the answer, would it not be reasonable to choose genotypes from a nicely parameterized distribution that is naturally defined on [0, 1] (e..g, the Beta distribution is flexibly-shaped and defined on the interval).

We thank the reviewer for raising this question. Yes, there is a truncation step applied to the new genotype values to make sure they are bounded between 0 and 1. We have made this clear in the pseudocode for our IBM, which can be found in the supporting information section. We appreciate the reviewer's suggestion to use a flexibly-shaped distribution, which we will definitely explore in our future work.

Lots of changes to Pd are described, first the phosphorus update, then a few additions or subtractions depending on cell loss, growth, death. Is it possible to give a complete formula (see above, for hi,n+1)? In terms of earlier comments about variable or fixed-length t, the mass gain described in Lines 79-80 suggests that the time step is fixed, otherwise I would expect mass gain (and other processes) to vary in magnitude or likelihood with t at each step. Can this be made more explicit? For example, if t does change, you might expect more growth over longer t and less growth over shorter t, so that the formula on Lines 79-80 would, again, depend on t. (Note: in the original ODEs this term wouldn't be expected to contain a t, but in the transition to a discrete-time framework it might (depending on details).

We thank the reviewer for these alternative suggestions to track change in P_d. However, since the time step is fixed indeed (see earlier comments), we maintain our original notation, with the additional clarifications about fixed time steps making the implications of the calculations we perform more clear. Also, we provide a detailed pseudocode in the supporting information section to enlighten the interested reader in exact procedures for computing the changes in individuals and dissolved phosphorous pool.

Line 108: If a host cell is infected, is it removed because it is assumed to die or because it can't be subsequently re-infected, and is no longer of interest dynamically? Are all bacteriophages lytic viruses (is that the implication)? Similarly, line 113 suggests that vi

represents the lone i th virion, which is lost to infection, but by chance mutations at earlier stages, other identical viruses could exist in the population, right?

Thank you for this valid question. Viruses in our model are indeed all modeled as lytic, with host death happening without any delay, i.e. dead host's biomass is immediately recirculated to the dissolved nutrient pool. It is also correct that a virus particle infecting a host is immediately removed from the pool of infective viruses, and indeed, other virus particles of equal traits may still exist in the infective pool of free viruses. We have added a specification on this in the main text.

Clarifications

I assume upon extinction/mutations that the population vector H could shrink or grow in length? I'm interpreting Nh as a dynamic quantity that measures the size of the system (in terms of total number of individual cells)? Same for V which could grow in length to 160K?

This is correct indeed, please see reply to comment above. **H** and **V** are indeed sets that grow (or shrink) as dynamics unfold.

Results

The results all show vertical axes of Nh and Nv which from Table 1 would be interpreted as total numbers of distinct cells. Is there any benefit to aggregating cells by genotype, or plotting measures of diversity? Would it be helpful to <u>quantify/present total diversity against time (if this isn't being what's shown) or biomass against time</u>? Since this is discussed as a tool for understanding <u>diversification and shaping diversity, the evolution of measures of diversity against time</u> (even simple ones) might make for compelling vertical axes in graphs.

We thank the reviewer for this suggestion and agree that evolution of diversity over time is one interesting outcome of our model. We now include a figure in the supporting information section showing evolutionary diversification in host and virus genotypes over time, using the Shannon entropy index. Including more details than that about diversification would in our opinion go beyond the scope of the present study. We are currently working on an in-depth analysis of trait-specific diversification, intended to be submitted as a follow-up study.

Presentation

Good attention is given to the presentation of the results in figures (in particular maintaining relatively consistent axes within and across figures, which facilitates comparison across results). I don't think the second panel of Fig 1, or either panel in Fig 2 are necessary. The formulas they present are relatively simple. Is there insight into whether the scale for the viral numbers is reasonable?

While we appreciate the reviewers comment on panels in Fig. 1 and Fig. 2, with trade-offs being a central aspect investigated in our study, we would like to keep these visual

illustrations of the trade-offs, especially for the sake of readers who are not very fluent in interpreting formula.

In my initial read, I missed the labels at the tops of columns in Figures 4-8, it would be nice if those could be moved into the figures, perhaps in legends. I think this would help consolidate information and make the reaction to the graphs more immediate.

We thank the reviewer for raising this concern. We modified the figures based on your comment to avoid any possible confusion. We removed the plot titles and added the labels inside the legends. We hope that these modifications improve the quality of the figures and make it easier for readers to follow the results.

It might be nice in the captions to include the baseline values of parameters not being varied (e.g., in Fig 4, what values of P and ω are used as V HR is being varied, and elsewhere, though I guess Line 162 addresses this). Also in Fig. 4, I'm not sure which panel(s) are being referred to with the phrase "Color codes for V HR match the codes in Fig. 3." since here there are more colors (and perhaps additional values) being plotted (the 3.30 line changes from orange to teal across figures, and a new 17.61 line appears).

Thanks for the comments but for readability, we prefer to not mention the default parameter values in each Figure. We think the figures would get too crowded. As noted correctly, we state in the text that parameter values are kept constant (default values shown in the table), except for those parameters that we vary, which are listed in the figure legends. We agree that since there is not an equal number of curves shown in Fig. 3 and Fig. 4, the reference to "matching color codes" was not suitable. We removed this sentence.

Again note captions mention t = 36 but the Table suggests this should be T = 36. Figure 6 caption is missing formatting on <u>y-axis</u> (i.e., y-axis, see line 166).

Thank you, we have corrected t to capital T in all figure texts. Regarding the y-axis formatting in Fig. 6, we have intentionally restricted labeling of y-axes and x-axes to subpanels on the left and bottom of the subpanel matrix, for the sake of visual clarity.

Is it possible to create a flow/transition diagram for the model itself (terms are presented, but not pseudo-code/equations so some features are hard to 'add up'). For example, The Pd equation (Line 71) seems like it has a sort of logistic growth feel to it (the increment depends on ω and is proportional to the difference between initial and present values). How does that relate to the within step increment/decrement to Pd via host cell dynamics? Is there normalization to ensure at the end of each 'step' that the total amount of dissolved phosphorous isn't violated? Also, on Line 88 is virus washout accounting for free virus in the solution that is washed out, or viruses associated with (i.e., inside) host cells that are removed as *host cells* are washed out? The detailed accounting would help tell the complete story of the interaction(s) in a more convincing way.

Thanks for the suggestions, we agree and provide a detailed pseudocode in the supporting information section for the interested reader to grasp procedural details about the workings of the model.

Is the interesting result mentioned on line 200 a consequence of the formula on line 71 (large washout removes cells - phosphorus, but then Pd is small, so a large term proportional to P 0 d − Pd gets added back?).

This is indeed an interesting observation and correct reasoning by the reviewer. High washout rates in chemostats (implying equal inflow rates) stimulate high growth in bacterial cultures (as long as washout rate does not exceed maximum growth rate in bacterial culture), due to large amount of supply of fresh resources from inflow. We now elaborate a bit on this in the discussion section.

Logistics and minor details

The Questionnaire suggests that code will be made available upon request. It would be a stronger commitment if it were posted to a public repository like GitHub or just directly as SI. Similarly no software language is referenced.

We thank you for your suggestion and concern. We added a reference to the project GitHub repository where the simulation code is made publicly accessible.

The sections lines 115-126 and Laboratory experiment are pretty brief, though the lab section seems reasonable (to a reviewer that doesn't do experiments). I wonder if more quantitative description of the model investigation is warranted in the SI (this may already exist, but I didn't see SI with the submission). For example, is the sensitivity analysis done according to any particular procedure, or is it a comparison across conveniently selected parameter values (is sensitivity defined in any formal sense?)? In Line 118 'A range of ... probabilities', is there a justification or rationale 3 for the range tested, perhaps characterize the parameters of the experiments here explicitly rather than deferring to figure/captions for details.

Thank you for the comment. We have performed sensitivity analyzes beyond those parameters presented in the text and include a figure in the supporting information section showing results of sensitivity analyses of additional host and virus trait parameters including host nutrient affinity, maximum host growth rate, burst size (number of viruses released upon successful infection) and viral adsorption coefficient. Generally, we varied parameters across a range of one to two orders of magnitude, covering realistic values for microbial systems. Typically, whereas the quantitative behavior of population dynamics was sensitive to choice of parameters, the qualitative behavior only changed gradually between the smallest and largest parameter values tested, giving us confidence that results are robust.

One larger issue here is the normalization of parameters as experiments are conducted. it seems like the Table 1 value for VHR (17.61) is the max tested, but the value for P (71) is intermediate. Some sort of normaiization scheme would help with the interpretation of dynamics.

We appreciate the reviewer pointing this out. Except for the VHR, we chose intermediate parameter values as default. We matched our default VHR of 17.61 with a value used in another study (Williams 2012, referred to in the text), but to allow comparison of our simulation results with results from our laboratory experiment (with VHR up to 3.3), we focused on testing diminishing VHR from that value downward. Please note that using an intermediate value for VHR in our study would not have changed results significantly, as results for VHR 3.3 and VHR 2 in our study are very similar to VHR 17.61 (please see Fig. 4).

Regarding normalization of output data, it was important for us to see the differences between the number of hosts and viruses in each plot, which are visible without normalizing the results. Hence, we prefer to keep the axes as they are now.

Line 101: consider a paragraph break at 'We also...' to enhance of the paragraph readability. Line 113: change 'anymore' to 'any more' Line 125: change 'parmeters' to 'parameters' Line 147: change to 20% (no space) Line 228: adsorption is misspelled Line 284: typo, first words in line Line 289: 'nutrient' not 'nutrientt' (two t's) All figure captions: verify T or t for simulation durations (I think ti should be T).

We thank the reviewer for pointing these out. We have made the suggested changes in the text.

Originality: This seems like a fairly original attempt at implementing an existing approach to bacteria-phage dynamics, based on a published continuous time, ODE model. Specifically novel bacterial chemostat is presented in parallel with model results.

Innovation: The paper addresses specific hypotheses about host-pathogen compatibility and virulence.

High importance to researchers in the field: The model is quite parameter rich (parameters mostly drawn from earlier theoretical model). Without a bit more detail on interpretation and implementation of a few key steps, there is a bit of uncertainty that might limit use by others.

We have addressed this concern by providing a pseudocode in the supporting information section, describing the steps and procedures in detail.

Significant biological and/or methodological insight: Despite framing the paper around mechanisms of diversity, most of the results are summaries of dynamics. So some of the opportunity for insight is undercut by the choice of results. It seems possible to focus directly on the diversity/coexistence angle by aggregating hosts and phage across genotypes.

Thank you for pointing this out. We conform to this suggestion by including and referring to results in the supporting information section, which summarize diversification of host and virus traits over time using the Shannon entropy index.

Rigorous methodology: There are a couple inconsistencies on the use of parameters (rates vs probabilities) and a bit of missing detail on implementation (time-step). Addressing this would make the model more convincing. I didn’t see reference to specific software used for implementation; this would facilitate reproducibility.

Thank you for pointing this out. We resolved the issue concerning rates and probabilities by clarifying that time steps are fixed 1 hour increments, giving an accurate translation from rates to “probabilities per time step” when rates have units "per hour”.

Substantial evidence for its conclusions: Conditions under which coexistence is possible are illustrated. Answers to questions (diversity) alluded to in the framing of the paper seem within reach and could make a more robust story. It seems like this little bit of extra computation/analysis would add quite a bit to the current results.

Thanks again for this suggestion, we agree and think that including the Shannon entropy as an index for diversification in a supplementary figure strengthens the manuscript.

Reviewer #3:

This paper investigates the hypothesis proposed by others (who are cited in the introduction) that a trade-off between bacterial host competitiveness and host defense against viruses will result in a persistent population of both virus and host. A model is described that takes into account mutation rate, nutrient availability and virus infection efficiency and the results compared to those of an in vivo experiment performed in E. coli with a T-4 like phage. The inclusion of the in vivo experiment is a major strengths of this work in my opinion.

The model parameters are clearly described in tables. The code is not included but is stated to be available upon request. Figures consist of graphs of parameters over time either in the live experiment or the model runs. Figures are generally clear, but there are ways they or their presentation could be improved.

The code is publicly available and we now link to the GitHub repository where it can be found. We thank the reviewer for suggestions to improve figures. We have added labels in the legends and included standard deviations based from 100 simulations as cloud error bars, showing the average of 100 simulations as solid line in each plot. We hope that these changes improve the quality of the figures, satisfy your expectations and make the results easier for readers to follow.

- **It is stated that the model is validated with laboratory data from a short-term infection experiment. However, the in vivo experimental result (Figure 3) and model result (Figure 4a) for host abundance differ considerably at the lowest VHRs and this does not seem to be addressed in the text.**

We appreciate the reviewer pointing this out. Indeed, some quantitative mismatch between low VHR in the laboratory experiment and our simulation results is apparent. It is possible that the discrepancy arises from the fact that VHR in our model is equivalent to the ratio of total number of *infectious* viruses to total number of hosts in our model, whereas in the laboratory system, not all viruses are infectious, such that only larger VHR in laboratory functionally correspond to VHR in our simulations. We comment on this in the discussion. Unfortunately, we do not have data on infectious particles available from the laboratory experiment. However, we also want to point out that although quantitative differences exist between the different VHR tested, the qualitative behavior between our simulation and laboratory experiment matches, with low VHR causing a later crash and later recovery of the host population.

- **Methods state that multiple repetitions were done, but no indication of variation between runs is indicated on the graphs and no raw data are provided.**

We thank the reviewer for the consideration and the comment. We considered the standard deviations as cloud error bars to show the variation between runs in the plots since as you mentioned we are reporting the average of 100 simulations for each plot. We hope that these changes improve the quality of the figures, satisfy your expectations and make the results easier for readers to follow.

The authors state clearly that their model is "highly idealized, focusing on specific trait-based mechanisms." However I found myself disagreeing with two assumptions that the investigation is based on, and this lessened my enthusiasm for the work.

Assumption 1: Evolution (genetic diversification) of bacteria requires the long-term co-existence of bacteria and phages.

While it is true that predator-prey relationships are a source of selective pressure that leads to genetic change over time, it is not the only source of selective pressure and therefore not necessary for evolution. Competition for nutrients, as included in the model, are just one of many other selective pressures. The fact that viruses exist and affect population dynamics of their hosts, to me, is not evidence that they are necessary for a healthy ecosystem.

We appreciate the reviewer's view and agree with their reasoning. Top-down control (be it predator or parasite driven) is not the only way to promote genetic change over time. Another example would be specialization for use of different resources (bottom-up control), which allows co-existence (and hence promotes diversity) over long time scales in the absence of top-down control. We also agree that existence of viruses and their influence on host population dynamics in itself does not provide any evidence that they are necessary for a "healthy ecosystem" (we have not used this term and it would have been helpful if the reviewer defined what is meant with "healthy ecosystem"). Besides that point, however, we never meant to claim that viruses are the only source of genetic diversification in the host (if this is what the reviewer means by "healthy ecosystem"). Instead, we tested whether interactions with viruses, in particular when they are regulated by trade-offs between competitive and defensive abilities in hosts and when they are random, can promote diversification in the host in an idealized system. Our simulations provide theoretical support for the hypothesis that non-random, trade-off regulated interactions are favorable for long-term co-existence, and we discuss our findings in terms of relevance for natural systems. But again, we have never claimed that this is the only mechanism, nor have we claimed that our results are proof that this is the most important mechanism at hand in nature. We have tried to be even more clear about this by including a new paragraph in the discussion reflecting what we state here, so as to avoid misleading conclusions that apparently could be perceived by readers.

Assumption 2: There is always a trade-off between the (bacterial) host's growth (competitiveness) and its abilities to defend against the virus. i.e. an increase in virus-resistance host will always result in slower reproductive rate.

If taken to the mathematical extreme, doesn't this assumption imply that a host with 100% virus resistance should be completely non-competitive with regard to growth? But absolute resistance to phage is known to exist in viable bacterial strains, which means in a single virus-host pair scenario, the virus would go extinct. Doesn't this disprove the single host-virus pair model?

We appreciate the reviewers view and agree that there is no proof that there always is a trade-off between the hosts competitiveness and abilities to defend against viruses (or predators for that matter). While there is some experimental support for such a trade-off, we have not meant to make this a universal claim in our study, and tried to be more clear in our formulations now, elaborating in a new paragraph in the discussion to avoid misleading conclusions.

We agree that absolute resistance to phage is known in viable bacterial strains, but to the best of our knowledge, this has only been shown in laboratory cultures, where competition is strongly limited compared to natural systems. In fact, Weinbauer (2004) formulated a "paradox" – why does viral infection seem ubiquitous in nature, whereas in laboratory conditions, bacterial strains readily evolve 100% resistance to viruses? A common interpretation of this "Weinbauer paradox" is exactly that in laboratory conditions, resistant strains do not have to compete with other strains. Interestingly, support of viral control being an important factor in determining competitive outcomes of bacterial taxa is also provided in experiments with natural communities where dominant taxa switched upon removal of viruses (Bouvier & del Giorgio). Yes, we agree, taken to the mathematical extreme, one may formulate 100% resistance to be equivalent with zero growth. There is no evidence for this in nature and it is not what we have based our study on. Note however that in a competitive setting with other bacteria, even only a partial reduction of growth as a consequence of 100% resistance will lead to extinction of the resistant host if the other competing hosts have higher growth rates, unless these other competing hosts are top-down controlled by viruses (or predators) (Gause's competitive exclusion principle).

References

Weinbauer MG (2004) Ecology of prokaryotic viruses. FEMS Microbiology Reviews, 28(2):127-181

Bouvier T & Del Giorgio PA (2007) Key role of selective viral-induced mortality in determining marine bacterial community composition. Environmental Microbiology, 9(2):187-297

minor typo: virues is missing an "r" in the abstract

Thank you for pointing this out. We have fixed the typo.

Have the authors made all data and (if applicable) computational code underlying the findings in their manuscript fully available?

The PLOS Data policy requires authors to make all data and code underlying the findings described in their manuscript fully available without restriction, with rare exception (please refer to the Data Availability Statement in the manuscript PDF file). The data and code should be provided as part of the manuscript or its supporting information, or deposited to a public repository. For example, in addition to summary statistics, the data points behind means, medians and variance measures should be available. If there are restrictions on publicly sharing data or code —e.g. participant privacy or use of data from a third party—those must be specified.

Reviewer #1: **No:** I did not see that the code for the simulations and the raw data from the laboratory experiment are made available.
Reviewer #2: **No:** Code is available upon request, but could probably be made available more immediately via SI or online repository.
Reviewer #3: **No:** The data points behind the plotted means and variance measures were not provided and there was no indication of a publicly accessible repository of the information or code.

We now provide the experimental data (experimental_data.csv) in the supporting information section and refer to the GitHub repository where the simulation code is made accessible.
