# Supplementary material for: Individual-based model highlights the importance of trade-offs for virus-host population dynamics and long-term co-existence
Source: PLoS Comput Biol. 2022 Jun 8;18(6):e1010228. doi: 10.1371/journal.pcbi.1010228 (PMC9212155; doi:10.1371/journal.pcbi.1010228)
Supplement: Attachment — Submitted filename: Response to Reviewers - Round 2.pdf [file pcbi.1010228.s007.pdf]

Response to Reviewer's comments – Round 2

We are very grateful for the second round of reviewers comments and are happy to announce that we addressed all comments, as outlined below (in bold font).

Reviewer #1: "I thank the authors for their detailed responses. Most issues were adequately addressed. The authors now include supplementary pseudocode, a GitHub link to their code, and a more detailed model description, all of which make it far easier for a reader to follow their methodology. The figures are also greatly improved. One minor remains issue with the figures: Fig 3-5 are missing an explanation as to the meaning of the shading."

Thank you for pointing this out, we have now added an explanation as to the meaning of the shading in the figure text.

"A key limitation that still exists is that phage population dynamics are not measured so that they cannot be directly compared to model results. In lines 284-287 of the discussion, this is touted as an advantage of the model. But I would argue that it is only an advantage if the viral population dynamics are independently verified with a rigorous comparison with data. Before such verification, the phage population dynamics seem speculative. In my view, such measurements are not needed for publication of this manuscript as the authors already state the challenges associated with obtaining such measurements. However, I do think that this limitation should be acknowledged."

Thank you for this valid comment. We agree and have reformulated the discussion to acknowledge the lack of experimental data on virus abundance as a shortcoming.

"Additional minor issue: Figure S2 does not appear to be referred to in the main text."

Thank you, we now refer to the results of Figure S2 in the main text.

Reviewer #2: "The authors seem to have addressed the majority of reviewer questions and concerns. While we might want a few more answers now, this paper seems to lay the groundwork for followup work.

I am satisfied with the revision and especially updates to the model description, tables/notation, and figures.

** I missed this the first time, but on line 145 a dot/bullet symbol is used in some chemistry notation. Not sure if this is standard."

Thank you for noticing this, we have now changed this to standard chemistry notation.
